# Optimistic Bias, Food Safety Cognition, and Consumer Behavior of College Students in Taiwan and Mainland China

**DOI:** 10.3390/foods9111588

**Published:** 2020-11-02

**Authors:** Guan-Yun Wang, Hsiu-Ping Yueh

**Affiliations:** 1 Department of Psychology, National Taiwan University, No.1, Sec 4, Roosevelt Rd., Taipei 10617, Taiwan; gywang.tp@gmail.com; 2 Department of Bio-Industry Communication and Development, National Taiwan University, No.1, Sec 4, Roosevelt Rd., Taipei 10617, Taiwan

**Keywords:** optimistic bias, social trust, information behavior, food safety, certification mark, purchase intention

## Abstract

The purpose of this paper is to investigate how optimistic bias, consumption cognition, news attention, information credibility, and social trust affect the purchase intention of food consumption. Data used in this study came from a questionnaire survey conducted in college students in Taipei and Beijing. Respondents in the two cities returned 258 and 268 questionnaires, respectively. Samples were analyzed through structural equation modelling (SEM) to test the model. Results showed that Taiwanese college students did not have optimistic bias but Chinese students did. The models showed that both Taiwanese and Chinese students’ consumption cognition significantly influenced their purchase intention, and news attention significantly influenced only Chinese students’ purchase intention. Model comparison analysis suggested significant differences between the models for Taiwan and mainland China. The results revealed that optimistic bias can be reduced in different social contexts as that of the Taiwan model and the mainland Chinese model found in this study were indeed different. This study also confirmed that people had optimistic bias on food safety issues, based on which recommendations were made to increase public awareness of food safety as well as to improve government’s certification system.

## 1. Introduction

FAO/WHO (Food and Agriculture Organization of the United Nations/World Health Organization) defined food safety risk management as conducting suitable undertakings, controlling the risk, and protecting public hygiene, and the primary goal of the management of risks associated with food is to protect public health by controlling such risks as effectively as possible through the selection and implementation of appropriate measures [1]. WHO further estimated of the global burden of foodborne diseases; the report suggested that, whether in developed or developing countries, about 10% of people worldwide will die from foodborne diseases. Therefore, effective risk communication to the public may be an important solution to improve people’s health [2]. Food safety is a cross-cutting issue related to human behaviors such as psychology, consumer behavior, and information technology [2,3,4]. In particular, the messages conveyed in risk communication can help people decide whether they can adopt some strategies to protect them from risk [2].

In 2014, food safety in Taiwan almost collapsed due to food scandals related to cooking oil containing recycled waste oil and animal feed oil. In 2016, some restaurants in mainland China illegally added opium poppy shell powder to hot pot, causing consumers to develop opiate addictions. As indicated by these major food safety incidents, although governments and food manufacturers should bear absolute responsibility, consumers must also have the wisdom to distinguish safe food.

Optimistic bias is one of the factors that affect people’s purchase intention [5]. Despite the numerous food safety incidents in Taiwan and mainland China, people remain optimistic about food consumption. This optimistic bias may be due to people ignoring the fact that everyone is in the same situation and has the same possibility of eating unsafe food; as a result, they believe they are less likely than others to face risks. People in Taiwan and mainland China generally do not trust their governments or the certification and authentication systems [6,7,8,9]. They are dissatisfied with the current state of food safety and tend to control it themselves. In other words, people think and act differently from the government policy regarding food safety, and they tend to take individual control by themselves with optimistic biased thinking.

This study aimed to identify the factors that affect college student’s consumption of certified food and agricultural products, and to understand their cognitive processes, including consumer awareness, information credibility, attention to news, and consumers’ social trust in government. These variables were examined through a model and further explored for policy making. In addition, this study attempted to compare the differences in the cognitive processes of food consumption of Taiwanese and Chinese college students under the impact of similar dietary habits and food safety incidents in response to different lifestyle, civil culture, and social systems.

## 2. Literature Review

### 2.1. Optimistic Bias and Food Safety Risks

Consumers tend to take responsibility and have the ability to avoid risk in food risk management [10]. Individuals may be able to assert that their chances of negative risks are less than average. However, it is practically impossible to prove that one is right. If everyone thinks they are at less than average risk, it could lead to systematic errors [11]. This error is called optimistic bias, which means that people tend to believe that they are less likely than others to encounter negative consequences, and that they are more likely than others to experience positive events [12]. Optimistic bias occurs when individuals compare the likelihoods that a risk event will occur to themselves and to others [11]. If the difference in the probability of assessing individual and others risks is greater, higher optimistic bias will result. Optimistic bias is essentially unrealistic optimism, which means that people tend to underestimate the likelihood of risky events, so they may not respond appropriately to those events [13].

Many studies in the field of social psychology or health communication have shown that optimistic bias exists [14]. Researchers have found that young people have higher self-efficacy in food safety, and they believe that they have sufficient knowledge to avoid risks [15,16]. In addition, research in Taiwan and mainland China has indicated that people have optimistic biases on public health issues such as bird flu outbreaks [16], prostate cancer screening [17], and the Influenza A virus subtype (H1N1) Vaccine [18]. Food safety, as the abovementioned issues, was also a risky problem in daily life for individuals, especially with many food safety crises happened in recent years. Although related studies supported that risk perception affected individual food purchase intentions and actual behaviors, further examination of how optimistic bias occurred when people perceived risky event was needed [19]. On the assumption that people have optimistic bias about food safety, previous studies suggested that people with higher safety awareness actually possessed purchasing intentions on safer food [20]. In order to facilitate individuals to make appropriate risk assessment, reducing the optimistic bias could be helpful to increase purchasing intentions.

### 2.2. Consumer Cognition of Certified Food and Agricultural Products

On the other hand, although consumers may have a neutral response to food safety issues, most of them agree that other people need more food safety advice than they do [21]. Generally, consumers worried about food safety of pesticides, antibiotics, and heavy metal pollution problems within the production and also worried about additives or industrial hazardous substances in food processing [22]. Consumers are the most important stakeholders in overall food safety management. Consumer cognition indicates consumer value, and this cognition is a key factor in the success of agricultural products with traceability markers [23]. Effective interventions should improve consumers’ risky food-handling behaviors via communication strategies or educational interventions [24,25], especially under consumers’ weak awareness of active prevention of food safety. Previous study supported that consumers seldom complain about food safety despite of their roles as the stakeholders involved and impacted by the problem [22]. Other studies have found that the source and certification of food access are the reasons consumers decide to buy food [6,20,26]. Therefore, it can be assumed that the attributes of certified foods will influence consumers’ purchase intentions because the certification mark provides some of the information that consumers expect.

### 2.3. Information Credibility and Attention to Food Safety

Information processing includes information attention, information access channel, and information source preference [9]. Researchers on risk communication of food safety have highlighted the importance of information accessibility, demand, and consumer-oriented risk perception [21,27,28]. The current study mainly focused on information attention and the credibility of access channels because certification marks may attract people’s attention and convince consumers that “certified products are safe.”

When people are aware of food safety risks, they need access to enough information to protect them, and the current food scandals have increased media attention on food safety. When people are exposed to this risk information, their concerns about food safety will be aroused, and they will temporarily change their behavior to mitigate risk [29,30]. If people have more demand for search information, they may take some steps to face the risk and try to solve the problem from the risk [31,32]. Therefore, this study assumed that if people pay more attention to food safety issues, they will be more willing to purchase certified food products.

Regarding the credibility of information, previous research mainly examined two aspects: information channels and information sources [9,31,33]. Wei et al. [32] pointed out that if the message is more credible, it can reduce optimistic bias and may change consumers’ behavior. Hu and Yueh [34] further proposed that the use of government information sources enhanced the credibility of risk information and reduced the public’s optimism bias. However, the public lacks incentives to report unsafe food to the government, and government regulations and policies need to be improved to further increase public awareness of food safety [35]. Therefore, consumer behavior and purchase intention depend on the credibility of the source of information, including regulations and government. The credibility of information is related to the legal effect and reputation [36,37,38]. Since the credibility of the information source reduces the uncertainty of the information seeker and increases the willingness to take risks, the credibility of the information may be an effective factor in consumer decision-making [17]. Therefore, there is reason to believe that if people think the information on food safety issues is more credible, they may be more willing to buy certified food.

### 2.4. Social Trust in Government in Food Safety Management

Risk management includes assessment, evaluation, and monitoring. The Taiwanese and mainland Chinese governments have both implemented certification systems to inform the public about food safety issues and to supervise food manufacturers. Most people tend to read food safety advice on food packaging [21]. In Taiwan and mainland China, when consumers want to understand the traceability of food, certification is the most important consideration because it can enhance consumer confidence and purchase willingness, and at the same time, it plays a role in educating consumers [8,39]. However, the reasons why consumers purchase certified food product rather than uncertified product are still called for further investigations. With reference to the compilation of cross-strait food certification marks by Liao et al. [33] and the food certification marks in mainland China introduced by Yin et al. [40], this study has compiled a comparison table for Taiwan and mainland China certification marks, as shown in Table 1.

Huang et al. [6] pointed out that although people lack sufficient background knowledge, they still like to see the government develop policies and encourage agricultural tourism. From the consumer perspective, they expect the government to provide a reliable certification mark to enhance consumer trust [41]. In addition, despite of the fact that consumers do not totally trust the government, they are still more willing to purchase government-certified products and trust the certification systems to a certain extent [20,42]. Previous study has found that the more consumers trust the food certification organization, the greater their willingness to purchase certified food [43]. Accordingly, the credibility and trust of government might be key points to consider when consumers buy food product.

Recent studies have also focused on the strategies and effects of regulators and governments in communicating with the public [38,44]. However, these studies have only discussed consumer or government issues separately, and a few have examined the relationship between consumers’ intentions to buy food and their social trust in government. Social trust raises individuals’ concerns about social issues and causes divisions in civil society [45]. Moreover, social trust also reflects people’s assessment of the structure of society, which is based on the individual’s knowledge that society consists of different groups having the same destiny.

Social trust can be measured by investigating government credibility. When the public lacks background information or is at a high potential risk, people will make decisions based on social trust [18]. If a food safety risk event occurs suddenly, people do not actually have enough information, which can also lead to a decline in government credibility. As suggested by Hu and Yueh [34], to improve risk communication, the government and media should value the interaction function of social media, reasonably encourage communication transparency, avoid providing incomplete information, and reduce the number of “unspoken secrets.”

Food safety issues are very important for college students; in particular, research has shown that a large proportion of them tend to eat out and therefore pay more attention to food safety incidents [46]. Although they may lack a positive attitude towards food safety practices and food safety [47,48], however, such students are also more flexible in accepting new things. Besides, well-educated young adults have higher acceptance of certified foods and more demand for consumer choice [8].

## 3. Materials and Methods

### 3.1. Hypothesis and Research Model

Based on the review of the literature, this study selected five concepts as the independent variables: optimistic bias; consumption cognition; information process, comprising news attention and information credibility; and social trust. From the perspective of optimistic bias, when consumers prepare food, their confidence in food safety behaviors is related to their strong belief in risk prevention [21]. Consumption cognition is related to what consumers will consider when purchasing food. Information processing includes attention to food safety news and the credibility of information sources and messages per se. Social trust is a public assessment of government credibility. Purchase intention of certified food product was selected as a dependent variable since it reflected the efficiency of governmental policies on food safety.

**Figure 1:**
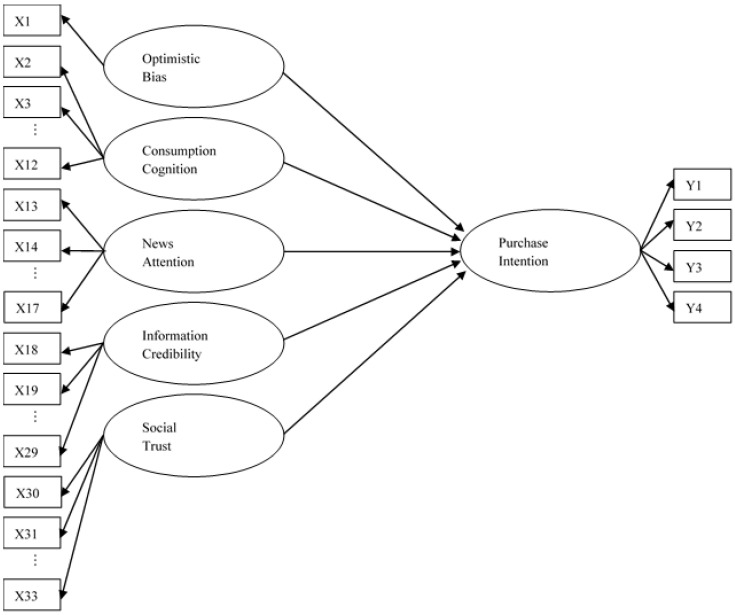
The conceptual model of food safety cognition and consumption attitude. Note: X1 to X33 and Y1 to Y4 refers to the sequential numbers of questionnaire items.

In sum, this study first examined college students’ optimistic bias (H1), and further considered a framework consisted of abovementioned variables. Figure 1 presents the model developed by this research, assuming the relationship between the above variables and purchase intention, and the interaction between these variables will be verified. The hypotheses for testing the model were as follows (H2–H6).

**Hypothesis 1** **(H1).** *Students have optimistic bias on food safety incidents*.

**Hypothesis 2** **(H2).** *Optimistic bias has a negative impact on purchase intention of certified food products*.

**Hypothesis 3** **(H3).** *Consumption cognition has a positive impact on purchase intention of certified food products*.

**Hypothesis 4** **(H4).** *News attention of food safety issues has a positive impact on purchase intention of certified food products*.

**Hypothesis 5** **(H5).** *Information credibility of food safety issues has a positive impact on purchase intention of certified food products*.

**Hypothesis 6** **(H6).** *Social trust has a positive impact on purchase intention of certified food products*.

Furthermore, this study compared models of food safety cognition and consumption attitudes among students in Taiwan and mainland China. Wilson et al. [38] conducted a cultural comparison study and found no significant differences in consumer perceptions of food safety regulators in Australia, New Zealand, and the United Kingdom. However, a study by Van Dijk et al. [44] found that communications with consumers, especially on food safety issues, may differ among cultures and countries.

Recent research on food safety issues in Taiwan and mainland China compares policies, government regulations, regulations, and certification marks [33,49], suggesting that social systems, cultures, and governments can cause different food safety issues and situations. Although Taiwan and mainland China have similar dietary habits (Chinese food), the two societies and civil cultures are different. To compare the two samples, this study tested the following hypothesis.

**Hypothesis 7** **(H7).** *There are differences in the two models of certified food purchase intentions for Taiwanese and mainland Chinese students*.

### 3.2. Data Distribution and Reliability

R 3.5.3 in RStudio 1.1.414 and LISREL 8.8 were initially used for analysis. This study consisted of multi-item scales of optimistic bias, consumption cognition, news attention, information credibility, social trust, and purchase intention. The study first checked that the data distribution conformed to the normality distribution. Items with skewness higher than 3 and kurtosis higher than 10 would be deleted.

### 3.3. Structural Equation Modelling

Using structural equation modelling (SEM) provides researcher a comprehensive picture of the theoretical framework [50]. The parameters of SEM used maximum likelihood estimation. The models were built and tested with a two-step paradigm [50] wherein the measurement model was tested first and the structural model was tested next. The measurement model was developed by confirmatory factor analysis. For determining the goodness of fit of CFA model, limiting the standard factor load value to between 0.5 and 0.95 is suggested [51].

Several indices were used to determine the goodness of fit of structural model. According to previous recommendations, normed chi-square (NC) (χ^2^/df) < 5 is acceptable, and <2 is a good model fit. As for root mean square error of approximation (RMSEA), <0.8 is good and <1 is an acceptable model fit [52]. In addition, confirmatory fit index (CFI) > 0.9 is a good model fit. If the analysis fails to meet the criteria for model fitting, the hypothetical model requires modification of alternatives.

### 3.4. Invariance Tests with Structural Equation Modelling

This study used SEM and the method proposed by Koufteros and Marcoulides [53] to perform two stages of invariance tests. For comparison, following Koufteros and Marcoulides [53], the invariance tests consisted of two phases, namely, the measurement model test and the structural model test.

Measurement equivalence can solve whether different groups of respondents interpret a given measure in a similar manner, so according to the suggestion of Vandenberg and Lance [54], the invariance of the measurement model was tested with 6 steps. After conducting the invariance of the measurement model, models can be ensured that they were measured in a similar way.

These 6 steps method will generate 6 models; in order to compare them, the χ^2^ differences would be tested in these 6 invariance test models. In addition, this study also considers the comparative fit index (ΔCFI), which represented in the results if it will be less than −0.01 or not. According to Cheung and Rensvold [55], for detecting cross-group differences in measurement invariance, values of ΔCFI < −0.01 indicate no significant differences between different invariance models.

To test the structural model invariance, the current study also followed the research method of Teng and Lu [56] to set the path coefficients from consumption cognition to purchase intention to be equal and freely estimated other path coefficients for the two samples.

### 3.5. Participants and Data Collection

Considering that urban residents have higher levels of knowledge and consumption than rural areas [8], their awareness of food safety knowledge and certified foods may be higher. This study was conducted using a purposeful sampling strategy, which targeted university students in two metropolises, Taipei and Beijing.

The respondents of this study were fully informed about the research purpose, and their participation was voluntary and autonomous. No additional ethical approval was required in this study, in accordance with national and institutional requirements. The numbers of completed questionnaires collected in Taipei and Beijing were 258 and 268, respectively, which satisfied a reliable samples size of 200 for SEM [57]. The survey included male and female students with different majors from different school year in the National Taiwan University and Peking University. The majority of respondents (92.6% in Taipei and 84.7% in Beijing) were aged between 18 and 23.

### 3.6. Measurement Scales of Variables

This study used multi-item scales to measure the major constructs, including students’ “optimistic bias,” “consumption cognition,” “news attention,” “information credibility” and “social trust” on food safety issues, as well as their “purchase intention.” Based on the theoretical framework shown in Figure 1, these scales were modified from previous studies, and all items of scales can be regarded as observed measures for the latent variables with 5 major constructs [50]. To test the “optimistic bias,” two kinds of judgments, i.e., comparative judgment and absolute judgment, were measured. For absolute judgment, numerical evaluation was used to find the relationship between self and personal experience [13]. Absolute judgment was evaluated with two questions: one to first assess the possibility that the “self” would experience risk and the other to assess the possibility that the “others” would experience risk.

This study referred to and revised the tools used by Lu et al. [31], Lu et al. [18], and Weng et al. [5] to measure optimistic bias. Two items were used to assess optimism, namely, “I think I myself may eat unsafe food” and “I think others may eat unsafe food” on a 6-point Likert-type scale (ranging from 1 = “strongly disagree” to 6 = “strongly agree”). Furthermore, the degree of optimistic bias was calculated by subtracting the self-optimistic evaluation value from the optimistic evaluation value of others. A higher score indicated higher optimistic bias.

The variables “consumption cognition,” “information credibility,” “news attention,” “information credibility,” and “social trust” were measured using a 6-point Likert-type scale (ranging from 1 = “strongly disagree” to 6 = “strongly agree”). The “consumption perception scale” consisted of 11 items and was a tool modified from Huang et al. [6]. The “news attention scale” consisted of 5 items and was modified from the scales developed by Lu et al. [18] and Wei et al. [32]. The “credibility of information scale,” modified from Lu et al. [46] and Wei et al. [32], was composed of 4 items related to message channels and 7 items related to message sources. The “social trust scale” contained four items revised with reference to the tools of Lu et al. [18]. Finally, the “purchase intention scale” contained 4 items, which were revised with reference to Lu et al. [18] and Weng et al. [5].

## 4. Results

### 4.1. Descriptive Statistics

The results of the analysis confirmed that all survey items were with skewness lower than 3 and kurtosis lower than 10. Table 2 provides the measurement items of the Taiwan and mainland China samples, the scale reliability (Cronbach’s α coefficient), and the factor load value. In this study, the reliability values of the subscales of the two samples were calculated, and the Cronbach’s α coefficients ranged from 0.7047 to 0.9481, indicating that the constructs included in the scale had high internal consistency.

This study checked the standard factor load value to between 0.5 and 0.95. Based on the analysis results, 5 items in the Taiwan questionnaire and 6 items in the mainland China questionnaire were deleted because they did not meet the loading score criteria. The factor loading values of all questionnaire items are listed in Table 2. After modification of the hypothesized items, all exogenous variables had good fit.

### 4.2. Identified Optimistic Bias

To determine whether students are optimistic about food safety incidents, this study proposed the hypothesis that respondents believe they are less likely than others to face this risk. Results of paired *t*-test analysis showed that respondents in Beijing believed the risk of eating unsafe food “themselves” (M = 4.83) was lower than that of “others” (M = 4.95) (t = −2.6977, *df* = 267, *p* < 0.01). However, Taipei’s respondents reported that there was no significant difference in the likelihood of “themselves” (M = 4.91) and “others” (M = 4.96) eating unsafe food (t = −1.3375, *df* = 257, *p* = 0.0911). Therefore, the H1 hypothesis was supported by the test results of mainland Chinese respondents but rejected by the test of Taiwanese respondents.

### 4.3. Hypothesis Testing of Taiwan Sample Structural Model

The hypothesized model included five latent exogenous variables (optimistic bias, consumption cognition, news attention, information credibility, and social trust) and one latent endogenous variable (purchase intention). This study used SEM to test the relationships between variables. In the Taiwan sample, the results of structural modelling analysis showed adequate fit to the data, as evidenced by χ^2^/df (NC) = 3.20, CFI = 0.91, and RMSEA = 0.093. However, the correlations between “optimistic bias” and other variables did not reach significance. Therefore, the correlation between “optimistic bias” and other variables was rejected, and an alternative model was constructed accordingly.

**Figure 2:**
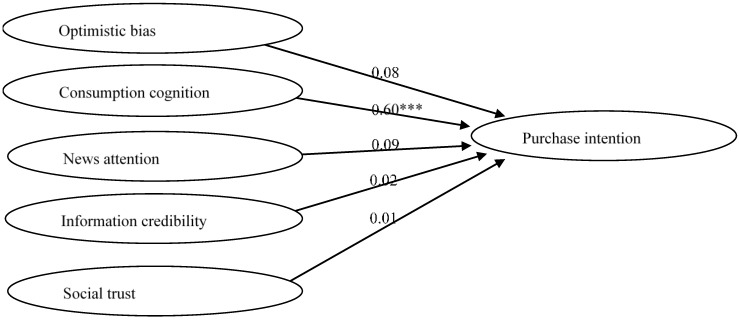
Path coefficient results of alternative model of the Taiwan sample. Note: *** *p* < 0.001; χ^2^ = 1348.26; df = 424; χ^2^/df (NC) = 3.17; CFI = 0.91; RMSEA = 0.092.

SEM analysis of the alternative model in the Taiwan sample indicated acceptable data fit: χ^2^/df (NC) = 3.17, CFI = 0.91 (≥0.9), and RMSEA = 0.092 (≤0.1). Table 3 presents the correlation coefficients between variables. As shown in Figure 2, the results of hypothesis testing demonstrated that only consumption cognition had a positive impact on purchase intention (β = 0.60, t = 8.41; *p* < 0.001); hence, H3 was supported. On the other hand, optimistic bias (β = 0.08, t = 1.55; *p* > 0.05), news attention (β = 0.09, t = 1.20; *p* > 0.05), information credibility (β = 0.02, t = 0.33; *p* > 0.05), and social trust (β = −0.01, t = −0.23; *p* > 0.05) did not predict purchase intention; therefore, H2, H4, H5, and H6 were all rejected.

### 4.4. Hypothesis Testing of Mainland China Sample Structural Model

In the mainland China sample, the results of structural modelling showed adequate fit to the data, as evidenced by χ^2^/df (NC) = 3.56, CFI = 0.94, and RMSEA = 0.098. As in the Taiwan model, no significant correlations between “optimistic bias” and any other variables were found, and an alternative model was constructed for further analysis.

**Figure 3:**
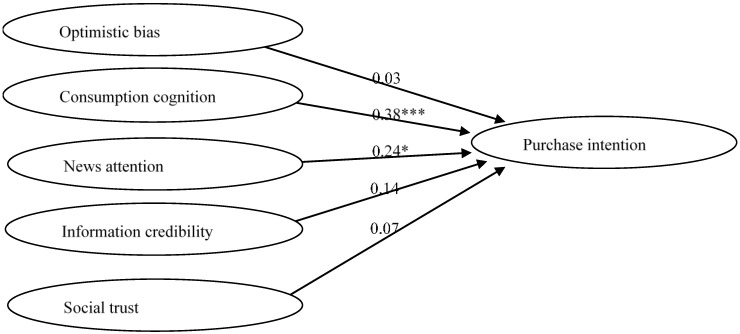
Path coefficient results of alternative model of the mainland China sample. Note: *** *p* < 0.001, * *p* < 0.5; χ^2^ = 1723.19; *df* = 485; χ^2^/df (NC) = 3.55; CFI = 0.94; RMSEA = 0.098.

SEM analysis results of the alternative model in the mainland China sample indicated acceptable data fit: χ^2^/df (NC) = 3.55, CFI = 0.94 (≥0.9), and RMSEA = 0.098 (≤0.1). Table 4 shows the correlation coefficients between variables. As shown in Figure 3, the results of hypothesis testing demonstrated that both consumption cognition (β = 0.38, t = 5.25; *p* < 0.001) and news attention (β = 0.24, t = 2.21; *p* < 0.01) had positive impacts on purchase intention; thus, H3 and H4 were supported. On the other hand, optimistic bias (β = −0.03, t = −0.60; *p* > 0.05), information credibility (β = 0.14, t = 1.59; *p* > 0.05), and social trust (β = 0.07, t = 1.08; *p* > 0.05) did not predict purchase intention; therefore, H2, H5, and H6 were all rejected.

### 4.5. Invariance Test for Comparison of Taiwan and Mainland China Models

For H7, it was hypothesized that differences in the two models of certified food purchase intentions would exist for the Taiwanese and mainland Chinese students. As shown in the previous model analysis, H3 was supported in both models; H4 was rejected in the Taiwan model but supported in the mainland China model; and H2, H5, and H6 were rejected. To verify the statistically significant differences, this study examined the degrees of invariance of the two models and then set up other fixed paths to calculate the difference in the causal coefficient of the H3 path.

Results of invariance of the measurement model analyses are shown in Table 5. To ensure that they were measured in a similar way, some items were removed because their variances or means were outliers.

Model 1 was used to test for configural invariance and freely estimate its factor loadings, factor variances, and covariance. Model 1 fits indices to the data RMSEA = 0.09820, NNFI = 0.92, and CFI = 0.93, and it was suggested to be plausible in all measurement situations. Next, model 2 tested metric invariance and suggested that the factor loading values of items were equal in the measurement scenario. Model 3 was a scalar invariance model; model 4 was used to test factor covariance invariance; model 5 was to test factor variance invariance; and model 6 was used for error variance invariance testing. After all analyses were performed in the LISREL program, the χ^2^ difference and the change in CFI values were calculated.

Although for the overall items in the Taiwan model and the mainland China models, the χ^2^ differences were significant in the invariance test models, the ΔCFI represented in the results was less than −0.01. According to the values of ΔCFI, therefore, the results showed that the invariance test of the measurement model was equal between the two groups and that the Taiwanese and mainland Chinese respondents in the study responded to the questionnaires in similar manners.

To test the structural model invariance, the current study also followed the research method of Teng and Lu [56]. As shown in Table 6, the causal coefficients of the baseline model of the two samples were freely estimated, and the path 2 model was constrained only by the causal coefficients of path 2, while other paths were freely estimated. According to the analysis results, the χ^2^ difference indicated that the causal coefficient of the H3 path in the Taiwan model was significantly different from that of the mainland China model (Δ$x^{2}$ = 4.4513, Δ*df* = 1, *p* = 0.03). Therefore, H7 was supported.

## 5. Discussion

### 5.1. Optimistic Bias of Taiwan and Mainland China

The reason why Taiwan college students did not have optimistic bias may be due to their personal life experience. Weinstein [11] pointed out that if people have experienced related risk events in past, they may increase the perceived likelihood that they will encounter risks, thus reducing the optimistic bias. In 2014, the news media in Taiwan reported food scandals related to edible oils, recycled waste oil, and animal feed oil. Cooking oil is involved in food preparation, and almost all foods consumed in daily life in Taiwan require the use of cooking oil. Unlike other past events, which only affected certain groups of people or certain kinds of food, this food safety crisis of edible oil, therefore, affected almost all the residents of Taiwan. This crisis led to a general perception among college students in Taiwan that their likelihood of encountering this risk was the same as the risk faced by everyone.

However, unlike college students in Taiwan, college students in mainland China had optimistic bias. This result showed that the students still had cognitive errors [11], and they tended to think that they were unlikely to encounter this risk. To reduce this optimistic bias, correct and sufficient information is highly needed. The intervention of education to make student know more about food safety issue can also improve students’ risk perception and confidence toward food [25].

### 5.2. The Differences of Structural Models between Taiwan and Mainland China

The Taiwan and mainland China models both significantly illustrated the causal relationship between consumption cognition and purchase intention, which echoed the findings of Huang et al. [6]. However, the results of this study are inconsistent with the findings of Elliott and Ellison [58]. They found that food safety awareness among middle school students is that the concept of food safety is equivalent to a food safety scandal. However, the results of this study found that from the perspective of consumer cognition, college students pay more attention to other aspects of food safety in addition to scandals.

Consumption cognition covers how people consider traceability, perceive the risk of food unsafety, and consume foods with organic labels and certification marks. The consumption cognition represented the value consumers place on food and affected their willingness to purchase certified food. Comparing the consumption models of college students in Taiwan and those in mainland China, the consumption cognition of Taiwan respondents had a greater impact on the causality of purchase intention. This result suggests that Taiwan respondents possessed better comprehension toward food consumption so that they relied on themselves and applied their own food knowledge autonomously. On the other hand, a previous study showed that people in mainland China cared more about the production date and shelf life of food product [59], which could result in weak purchase intention of certified food. However, the results of the study also suggested the needs of more active measures taken by the governments and manufacturers to increase consumer’s inherent awareness of food selection, preparation, handling, and even cooking and eating. For the college students in both Taiwan and mainland China, imparting more knowledge about food to increase awareness may cause people to value food and make them want to buy certified food. Despite some gaps in knowledge about certified food, people are still willing to buy it and are motivated by health and environmental reasons [42].

Another difference between Taiwanese and mainland Chinese college students was the impact of news attention on purchase intention. In Taiwan, there was no significant causal relationship between news attention and willingness to buy, but news attention had an effect in mainland China. People are eager for more information because they are aware of the risks and want to take actions to reduce their uncertainty about the risks [60]. However, young adults often lack the knowledge or motivation to use their ability to solve food safety issues, and this information behavior is temporary [15,30]. To develop lasting habits, this study considered that education on food media literacy and drawing attention to food safety issues are very important. In addition, mobile media have received a lot of attention in mainland China, especially because information on the food scandals was widely disseminated by the public on social media [61], and most users take complete advantages of the multimedia and interaction functions available on social media [34]. Such new media types might continue to remind people to pay attention to food safety.

In both the Taiwan and mainland China samples, the causal effects of information credibility and social trust on purchase intention were not significant. Lu et al. [18] pointed out that if the news media continue to expose negative scandals about these events and if the government does not explain them in due course, the public will no longer trust the government. Therefore, the results of this study indicated that neither the Taiwanese nor the mainland Chinese government has responded appropriately on food safety issues. Because government-certified foods lack the public’s social trust, people do not consider information about food and social trust when deciding whether to buy food with a certification mark. To make communication more effective, regulators need to reduce consumer-perceived information overload, provide targeted consumers with the right information, and reduce the gap between consumers and regulators [10,38].

## 6. Policy Implications

### 6.1. The Development of Food Certification Marks

Since 2013, the State Council of the People’s Republic of China has formulated 11 regulations and several documents on food safety. In 2015, the “Food Safety Law” was promulgated and implemented. To improve risk management of food safety, the government tried to revolutionize the organizations [62]. Since there are several related agencies responsible for food safety [63], the government has attempted to cooperate with these agencies. Overlapping government agencies include the Ministry of Commerce, the Ministry of Industry and Information Technology, the Ministry of Public Security, the Ministry of Agriculture and Rural Affairs, the Food and Drug Administration, the Quality Supervision Administration, and the National Security Administration [7].

On the other hand, after the cooking oil scandal in Taiwan in 2014, the public’s anger at Taiwan’s harsh food safety environment reached its highest point. As a result, the Taiwanese government established the “Food Safety Office of the Executive Yuan” to help liaise with interministerial food safety policies including the Agricultural Committee, Environmental Protection Agency, and Ministry of Health and Welfare. At the same time, the Taiwan government formulated the “Act Governing Food Safety and Sanitation” at the end of 2014 as the highest guiding law for the management of food safety and hygiene.

Section 2.4 of this paper summarizes the certification marks currently used by both governments. The legal sources of food certification marks in mainland China include the Regulation of the People’s Republic of China on the Administration of Production License for Industrial Products and the Trademark Law of the People’s Republic of China. In order to make it easier for consumers to choose safe food, the Taiwan government has issued about 20 different certification marks. These certification marks have different legal sources and are issued by different units. It includes Agricultural Production and Certification Act, Agricultural Food Control Act, Act Governing Food Safety and Sanitation, etc.

In addition, in the context of promoting cross-strait exchanges, in November 2008, the Taiwan government and the Chinese government signed the “Cross-Strait Food Safety Agreement”; the purpose is to enhance cross-strait food safety communication and mutual trust. This agreement was negotiated between the Straits Exchange Foundation and the Association for Relations across the Taiwan Straits.

### 6.2. Food Safety Incidents and Government Response

Over the past 20 years, there have been many serious food safety incidents in mainland China and Taiwan. In China, in 2008, melamine-contaminated milk powder caused babies with kidney stone disease. The milk powder producer Sanlu also lost public trust [63]. In 2010, another gutter oil scandal occurred in China. Between 2005 and 2011, news reports indicated that three leather milk incidents occurred. Milk manufacturers use waste leather to squeeze leather protein powder and add it to milk to increase the ratio of milk protein. In 2015, “zombie meat” was illegally imported into China, and these meats are all frozen meat that has expired. As food delivery services have become more and more popular in recent years, many related food safety scandals have also appeared in 2016.

Faced with so many food scandals, starting from 2011, the Food Safety Committee of the State Council of China held the “China Food Safety Promotion Week” every June. The project is seen as an educational activity for the public to popularize scientific knowledge and improve public perception of risk. Until 2019, it has been held about 8 times. In addition, at the end of 2019, the new coronavirus disease affected the entire world. Under such circumstances, food safety has also become a worrying issue. In China, traditional eating habits may increase the possibility of infection. In particular, February 2020 is the Chinese New Year, and the family’s return to their hometowns also makes the virus more likely to spread more quickly. Therefore, the habit of eating together at the table should be different from the past [64].

As for Taiwan, in 2009, a large number of ducks contaminated with dioxin died. In 2011, unscrupulous manufacturers used cheap industrial plasticizers to replace normal food additives. Food and beverages containing plasticizers such as bis(2-ethylhexyl) phthalate (DEHP) were sold in stores. These foods caused harm to the human body, affecting the people not only of Taiwan but also of Hong Kong and China. In 2014, cooking oil recovered from restaurant waste almost destroyed many food manufacturers in Taiwan. In 2015 and 2016, there were also scandals of unsafe food and restaurants selling food with pesticides. As a result of the food security scandal in 2014, the Taiwanese government has established important organizations and government agencies since then, and also formulated special laws to manage it.

The perpetrators of the most serious food safety scandals in mainland China and Taiwan have been sentenced to heavy penalties. Taiwan Dingxin Enterprise has been fined US $3 million, and the chairman was sentenced to 22 years in prison. Sanlu, Zhang Yujun, and Geng Jinping of mainland China have all passed away [65].

### 6.3. The Role of Social Trust and Information Credibility

From the foregoing discussion, it can be seen that the government plays an important role in dealing with food safety issues. In spite of the availability of more useful information, still the general public may be subjective and the information would be inefficient [20]. Therefore, if it can increase the public’s trust in the government and the credibility of disseminating information, it should be beneficial to public communication and education efficiently [20,25]. Liu et al. [66] also indicated that to raise public concern, one cannot just reply in the media. Although the media continues to report food safety news, it still has no significant relationship with the public’s concern. However, the government needs to pay more attention to the public’s views and make the public more concerned about food safety. Meanwhile, it is necessary to strengthen risk communication between the government and the public, and in the future, the news media should be used in more efficient way [22].

Food safety labeling and certification systems can be a simple and effective way to inform consumers that food products with a mark are safe and trustworthy [7,20]. In order to make certification more reliable, it is necessary to trace back to the public’s trust in the government’s supervision of food safety. The model proposed by this current research takes into account the purchase intention of certified food and thus can help to understand the relationship between consumer consumption decisions and government policies.

Liu et al. [63] recommended that public authorities need to provide the public with detailed information such as traceability information, food certification, and other food quality standard information. The results of this study showed that people tend to focus on their consumer perceptions and there is no significant relationship between media attention and information. The result also echoed Zhang et al. [48], which indicated that the more the information access people have, the less chances they would examine food safety. It is recommended that the government need to make more efforts in the future to maintain its credibility and provide more reliable information in order to facilitate more positive and appropriate public perception of food safety.

## 7. Conclusions

This study proposed factors that influence the purchase intention of certified foods, including optimistic bias, consumption cognition, news attention, information credibility and social trust. The results of this study revealed that students’ optimistic bias can be reduced in different social or cultural contexts and that the Taiwanese model and the mainland Chinese model proposed in this study were indeed different. Through empirical research, the results confirmed once again that people do have optimistic bias on food safety issues. College students tend to think that they are less likely to eat unsafe food than others, and this bias becomes a systemic bias. The results show that the optimism bias of the two groups of students is different, which may be due to differences in personal experience and social context, leading to different degrees of cognitive biases on food safety between Taiwanese students and mainland Chinese students.

In this study, consumer cognition significantly affected the willingness of Taiwanese and mainland Chinese college students to purchase food with certification marks. News attention significantly influenced only the willingness of mainland Chinese students to purchase certified food, not that of Taiwan students. Meanwhile, the other variables in the two models had no significant relationships with purchase intention. The results of this empirical study showed that people tended to believe in themselves and isolated themselves from other sources of information. In addition, consumers valued the product itself, indicating that their consumption perception comes from the consumption process. For this reason, it is difficult for the media and government to exert influence on consumers. In addition, the media can have a small impact on consumer behavior only when consumers are actively concerned about the issue. Therefore, it is necessary to improve consumers’ impressions of products, their governments and even media resources. The quality of news from the government, the media and manufacturers should also be improved.

Since data were collected only from Taipei and Beijing, which is an important limitation of the study, it must be noted that the results cannot be generalized to other populations. However, this empirical study also specifically discusses the impact of social context. The governments in Taiwan and mainland China implemented different measures on food safety management, certification marks, and policy communication, so college students on the two sides of the Taiwan Strait showed different purchasing behaviors in the two models. The different information behaviors adopted by college students in Taiwan and mainland China may cause them react differently to food risk events. It is recommended that future research should re-examine the impact of government policies, and to what extent consumers may only believe in themselves, rather than rely on the credibility and trust of the government and food manufacturers.

Furthermore, people’s attitudes towards food safety issues may change over time, and more social context variables may need to be considered. In this current study, the food safety cognition mainly focused on people’s views on food safety and their information processing behavior. While the subjects recruited were only college students, it is suggested that future research can be expanded to include a wider sample or consider more variables to improve the generalizability of the model. Moreover, this study found that optimistic bias was not the main factor affecting purchase intention, and it showed different results in the two models of college students in Taiwan and mainland China. This weak explanatory power reflects the need to explore other food safety risk assessment factors. Future research should consider time effects, subject areas, and other risk assessment variables, and more psychological and social factors can be added to better understand food safety risk assessment issues.

## Figures and Tables

**Table 1:** Common certification marks in Taiwan and mainland China.

Taiwan		Mainland China	
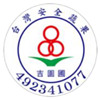 Good Agricultural Practice, GAP	This mark represents that the product contains allowable levels of pesticides and that farmers and the public are taught about proper pesticide usage.	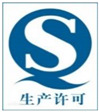 QS mark	“QS” is the abbreviation of “Quality and Safety.” In mainland China, all foods sold on the market should carry this mark.
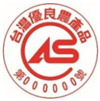 Certified Agricultural Standards	This mark indicates that agricultural products meet Taiwan’s agricultural standards.	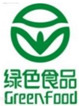 Green Food	This symbol indicates that agricultural products are grown in an environment-friendly manner with less pollution and good quality.
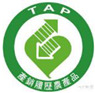 Traceability Agricultural Product, TAP	This mark means that agricultural products can be traced back to their production processes and are environmentally friendly.	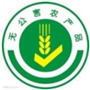 Harmless Agricultural Product	This mark indicates that the pollution content of agricultural products is within the safe range and meets standards.
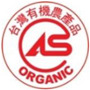 CAS organic agricultural Product	This mark refers to organic products that meet standards during processing.	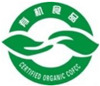 Organic Food	This mark refers to organic foods and confirms the absence of artificial materials, genetic engineering and environmental pollution during the production process.

**Table 2:** Descriptive statistics and confirmatory factor analysis of scale items in food safety (Taiwan, N = 258; mainland China, N = 268).

Statement	M ^1^ (T)	M (C)	SD (T)	SD (C)	Factor Loading (T)	Factor Loading (C)
Optimistic bias ($\alpha_{T}=\;0.9021$, $\alpha_{C}=0.9281$) ^2^						
I think I myself may eat unsafe food.	4.91	4.83	0.92	1.42	–	–
I think others may eat unsafe food.	4.96	4.95	0.96	1.36	–	–
X1. “others” minus “self”	0.05	0.12	0.56	0.72	–	–
Consumption cognition ($\alpha_{T}=\;0.8765$, $\alpha_{C}=0.9050$)						
I value						
X2. food products having certification	3.90	4.28	1.11	1.62	0.71	0.71
X3. the package	4.60	4.40	1.17	1.53	0.65	0.70
X4. the expiration date	5.33	5.51	0.88	1.16	–	–
X5. food products having safe and hygienic marks	4.57	4.62	1.04	1.56	0.66	0.83
X6. food products made in my nation	3.52	2.88	1.18	1.46	0.64	–
X7. place of production	3.88	3.14	1.21	1.51	0.64	–
X8. freshness	5.15	5.24	0.92	1.25	0.57	0.67
X9. food products being contaminated	4.88	4.64	1.05	1.52	0.54	0.85
X10. food products having pesticide residue	4.48	4.45	1.14	1.58	0.66	0.82
X11. the record of production	3.74	3.61	1.13	1.60	0.74	0.69
X12. the manufacturers	3.97	3.75	1.19	1.66	0.63	0.56
News attention ($\alpha_{T}=\;0.7353$, $\alpha_{C}=0.8358$)						
I pay attention to food safety news on…						
X13. newspaper	3.54	2.99	1.45	1.63	0.72	–
X14. television	4.09	3.74	1.30	1.63	0.80	0.80
X15. the Internet	4.76	2.68	1.07	1.55	0.58	0.60
X16. radio	2.59	4.56	1.38	1.49	0.53	0.77
X17. mobile phone (e.g., scanning the QR code)	3.10	3.59	1.53	1.63	–	0.60
Information credibility ($\alpha_{T}=\;0.9155$, $\alpha_{C}=0.9481$)						
Information about food safety on/from…is credible.						
X18. newspapers	3.61	3.40	1.09	1.40	0.90	0.87
X19. television	3.48	3.44	1.05	1.41	0.94	0.89
X20. the Internet	3.38	3.21	1.01	1.33	0.76	0.87
X21. radio	3.38	2.87	1.11	1.27	0.84	0.79
X22. mobile apps	3.39	3.12	1.03	1.33	0.78	0.79
X23. media	3.28	3.13	1.10	1.33	0.81	0.83
X24. government	4.05	3.85	1.14	1.45	–	0.74
X25. research institutes	4.55	4.31	0.99	1.44	–	0.74
X26. consumer protection agency (NGO)	4.34	2.99	1.02	1.32	0.52	0.77
X27. manufacturers	3.00	2.76	1.09	1.30	–	0.68
X28. family members and friends	2.94	3.14	1.20	1.40	0.51	0.64
X29. Facebook pages/WeChat official accounts	2.65	2.64	1.11	1.24	0.57	0.68
Social trust ($\alpha_{T}=0.7047$, $\alpha_{C}=0.8151$)						
I think governments…in/on food safety.						
X30. are correct to adopt policies	3.57	3.97	1.11	1.49	0.86	0.85
X31. are credible to adopt policies	3.40	3.59	1.14	1.41	0.96	0.90
X32. are able to solve problems	3.31	3.70	1.30	1.48	0.65	0.67
X33. should develop long-term plans	5.15	5.28	1.06	1.33	–	0.51
Purchase intention ($\alpha_{T}=0.9150$, $\alpha_{C}=0.9444$)						
I tend to…food products which have certification marks.						
Y1. buy	4.74	4.68	1.09	1.58	0.97	0.96
Y2. eat	4.80	4.75	1.07	1.52	0.96	0.97
Y3. increase instances of buying	4.66	4.67	1.13	1.55	0.78	0.85
Y4. pay more money to buy	4.19	4.30	1.20	1.61	0.68	0.76

^1^ The abbreviations used in this table and throughout this paper are as following: M = mean and SD = standard deviation. In the table, Taiwan also was abbreviated T, whereas mainland China was abbreviated C. ^2^ The value of α indicated Cronbach’s alpha. Taiwan was abbreviated T, whereas mainland China was abbreviated C. In addition, X1 to X33 and Y1 to Y4 indicated the sequential numbers of questionnaire numbers. Measures of latent exogenous variables represented X, whereas measures of latent endogenous variables represented Y.

**Table 3:** Correlation coefficients between variables in Taiwan sample model.

	Optimistic Bias ^1^	Consumption Cognition	News Attention	Information Credibility	Social Trust
Optimistic bias	1.0000				
Consumption cognition	0.0338	1.0000			
News attention	−0.0914	0.3721 *** ^2^	1.0000		
Information credibility	−0.0429	0.2809 ***	0.4468 ***	1.0000	
Social trust	0.0447	0.2554 ***	0.1744 **	0.3395 ***	1.0000

^1^ In the alternative model, the correlation relationships between optimistic bias and other variables were rejected. ^2^ *** *p* < 0.001, ** *p* < 0.01.

**Table 4:** Correlation coefficients between variables in the mainland China model.

	Optimistic Bias ^1^	Consumption Cognition	News Attention	Information Credibility	Social Trust
Optimistic bias	1.0000				
Consumption cognition	−0.0068	1.0000			
News attention	−0.0459	0.5580 *** ^2^	1.0000		
Information credibility	−0.0878	0.4170 ***	0.6359 ***	1.0000	
Social trust	−0.0968	0.4575 ***	0.5278 ***	0.5740 ***	1.0000

^1^ In the alternative model, the correlation relationships between optimistic bias and other variables were rejected. ^2^ *** *p* < 0.001

**Table 5:** Six steps of measurement invariance test between the two models.

Model ^1^	χ^2^	Δχ^2^	df	Sign. Level	RMSEA	NNFI	CFI	ΔCFI
1. Configural Invariance	1564.0978	–	486		0.09820	0.9215	0.9309	–
1 versus 2	–	41.4246	–	0.000	–	–	–	−0.0014
2. Metric Invariance	1605.5224	–	505		0.09730	0.9229	0.9295	–
2 versus 3	–	144.0124	–	0.000	–	–	–	−0.0054
3. Scalar Invariance	1749.5348	–	528		0.9870	0.9207	0.9241	–
3 versus 4	–	48.2809	–	0.000	–	–	–	−0.0024
4. Factor Covariance Invariance	1797.8157	–	538		0.09930	0.9197	0.9217	–
4 versus 5	–	36.4852	–	0.000	–	–	–	−0.0015
5. Factor Variance Invariance	1834.3009	–	533		0.1008	0.8930	0.9202	–
5 versus 6	–	136.8797	–	0.000	–	–	–	−0.0094
6. Error Variance Invariance	1971.1806	–	552		0.1047	0.9108	0.9108	–

^1^ χ^2^ represents chi-square. df represents degree of freedom. Sign. Level represents significant level (α = 0.05). RMSEA represents root mean square error of approximation. NNFI represents Non-normed Fit Index. CFI represents Comparative Fit Index. Δ represents the differences.

**Table 6:** Invariance test of the two-group structural model.

Model	χ^2^	df	Δχ^2^	Δdf	Significance Level
Baseline model	1377.669	432			
Consumption cognition → Intention model	1382.12	433	4.4513	1	0.03 *

χ^2^ represents chi-square, df represents degree of freedom, and Δ represents the differences. * *p* < 0.05.
